# Efficiency of compensatory orthodontic treatment of mild Class III malocclusion with two different bracket systems

**DOI:** 10.1590/2177-6709.22.6.049-055.oar

**Published:** 2017

**Authors:** Mônica L. C. Aragón, Lívia M. Bichara, Carlos Flores-Mir, Guilherme Almeida, David Normando

**Affiliations:** 1 Private practice (Belém/PA, Brazil).; 2 University of Alberta, Department of Dentistry, Division of Orthodontics (Edmonton, Canada).; 3 Universidade Federal de Uberlândia, Faculdade de Odontologia, Departamento de Ortodontia (Uberlândia/MG, Brazil).; 4 Universidade Federal do Pará, Faculdade de Odontologia, Programa de Pós-graduação em Odontologia (Belém/PA, Brazil)​.

**Keywords:** Orthodontic appliances, Orthodontic brackets, Malocclusion, Angle Class III

## Abstract

**Objective:**

The purpose of this study was to assess the efficiency of compensatory orthodontic treatment of patients with mild Class III malocclusion with two preadjusted bracket systems.

**Method:**

Fifty-six matched patients consecutively treated for mild Class III malocclusion through compensatory dentoalveolar movements were retrospectively evaluated after analysis of orthodontic records. The sample was divided into two groups according to the brackets used: Group 1 = non-Class III compensated preadjusted brackets, Roth prescription (n = 28); Group 2 = compensated Class III preadjusted brackets, Capelozza III prescription (n = 28). Cephalometric analysis, number of appointments and missed appointments, months using Class III elastics, and bond/band failures were considered. Treatment time, Peer Assessment Rating (PAR) index at the beginning (PAR T_1_) and end of treatment (PAR T_2_) were used to calculate treatment efficiency. Comparison was performed using a MANOVA at *p*< 0.05.

**Results:**

Missed appointments, bond or band failures, number of months using the Class III intermaxillary elastics, and cephalometric measurements showed no statistically significant difference (*p*> 0.05) between groups. Patients treated with Roth brackets had a treatment time 7 months longer (*p*= 0.01). Significant improvement in the patient’s occlusion (PAR T_2_-T_1_) was observed for both groups without difference (*p*= 0.22).

**Conclusions:**

Orthodontic brackets designed for compensation of mild Class III malocclusions appear to be more efficient than non-compensated straight-wire prescription brackets. Treatment time for Class III patients treated with brackets designed for compensation was shorter than with Roth prescription and no difference in the quality of the occlusal outcome was observed. A prospective randomized study is suggested to provide a deeper look into this subject.

## RESUMO

**Objetivo:**

o objetivo desse estudo foi avaliar a eficiência do tratamento ortodôntico compensatório de pacientes com má oclusão de Classe III suave usando dois diferentes sistemas de braquetes pré-ajustados.

**Métodos:**

foram avaliados retrospectivamente, após análise de registros ortodônticos, cinquenta e seis pacientes tratados consecutivamente de má oclusão de Classe III, por meio de movimentos de compensação dentária. A amostra foi dividida em dois grupos, de acordo com os braquetes utilizados: Grupo 1 - braquetes pré-ajustados não compensatórios para Classe III, prescrição Roth (*n* = 28); Grupo 2 - braquetes pré-ajustados para tratamento compensatório de Classe III, prescrição Capelozza Padrão III (*n* = 28). Considerou-se a análise cefalométrica, número de consultas realizadas e de consultas perdidas, meses de uso dos elásticos intermaxilares de Classe III e quebras de braquetes/bandas. Foram utilizados para calcular a eficiência do tratamento: tempo de tratamento, índice PAR (*Peer Assessment Rating*) ao início (PAR T_1_) e fim de tratamento (PAR T_2_). A comparação intergrupos foi realizada com o teste MANOVA, a *p*< 0,05.

**Resultados:**

não houve diferença estatisticamente significativa (*p*> 0,05) entre os grupos quanto às medidas cefalométricas, número de consultas perdidas, quebras de braquetes/bandas e tempo de uso dos elásticos de Classe III. Os pacientes tratados com braquetes Roth tiveram tempo de tratamento sete meses maior (*p*= 0,01). Observou-se melhora significativa na oclusão dos pacientes (PAR T_2_-T_1_) para ambos os grupos, sem diferença estatística significativa (*p*= 0,22).

**Conclusão:**

os braquetes ortodônticos projetados para compensação das más oclusões de Classe III parecem ser mais eficientes do que os pré-ajustados não compensatórios. O tempo de tratamento para pacientes Classe III tratados com braquetes projetados para compensação foi menor do que com a prescrição Roth, e nenhuma diferença na qualidade da oclusão final foi observada. Sugere-se um estudo prospectivo randomizado, para fornecer uma visão mais profunda sobre esse assunto.

## INTRODUCTION

Since the introduction of preadjusted orthodontic appliances the number of prescriptions has significantly increased.^1^ Mesiodistal angulation (tip) and buccolingual inclination (torque) for specific tooth types are the main features differentiating orthodontic bracket pre-adjusted prescriptions. Some are designed to treat malocclusions that require specific compensatory movements. However, in most cases, these prescriptions are introduced to the orthodontic market without proper assessment of their efficiency, in other words, good results in less time of treatment 

Class III malocclusions have a low prevalence in Western populations^2^ and higher among Chinese and Malaysian populations.^3^ moderate to severe Class III malocclusions can exert considerable impact on patient’s aesthetics^4^ and quality of life.^5^ In most cases, they exhibit natural dental compensations whereby maxillary canines appear mesially angulated while maxillary incisors manifest an increased labial inclination.^6^ Simultaneously, mandibular canines appear more upright with the mandibular incisors more lingually inclined.^7^ ^-^ ^9^

**Figure 1:**
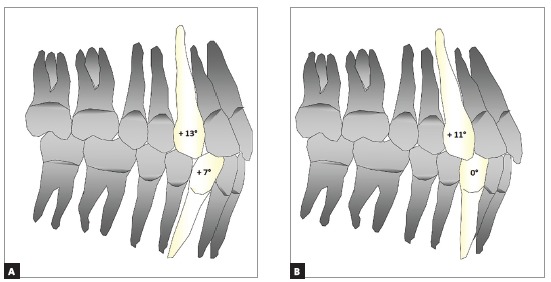
Illustrative simulation of the influence of canine angulation on incisor positioning. A) Maxillary canines appear mesially angulated, and lower canines more angled, increasing inclination of the central incisors and the perimeter of the arch - Roth prescription. B) Maxillary canines exhibit dental compensation, more angulated, while lower canines less angulated, decrease inclination of the incisors, facilitating the torque compensation applied to the central incisors in Class III patients - Class III compensated brackets prescription.

In 1999, a system of individualized preadjusted brackets was introduced with built-in angulations and inclinations in their slots varying according to the required dentoalveolar compensation type.^8^ According to the developers of this system, the compensatory strategy in treating Class III malocclusions should involve preservation or enhancement of preexisting natural compensations. The author drew from an original idea proposed by Andrews,^1^ a decade before, who suggested that different torques should be applied to incisors according to the nature of the malocclusion to be compensated. Further canine mesiodistal angulation was introduced with the purpose of optimizing incisor compensation.^8^ The ultimate goal of these modifications was to increase the length of the maxillary arch while decreasing the length of the mandibular arch.^9^ ^,^ ^10^ Conventional straight-wire bracket systems such as Roth are characterized by lower anterior brackets with mesial tip that procline these teeth, therefore making it difficult to compensate Class III malocclusions (Fig 1). Thus, the flaring of the anteroinferior teeth could not be avoided when using non-compensated prescriptions.^1^ ^,^ ^11^ ^,^ ^12^

Despite the theoretical framework behind this bracket prescription, no clinical study has evaluated the actual clinical efficiency of this treatment option. Therefore, the aim of this study was to investigate the efficiency of compensatory orthodontic treatment of patients with mild Class III malocclusion with a preadjusted bracket system designed for compensation, compared to Roth prescription brackets.

## METHODS

This study was approved by the Ethics in Research Committee (CEP-ICS / UFPA) registered under number 517.398. This retrospective study evaluated consecutively treated mild Class III malocclusion cases treated through compensatory dentoalveolar movements. Orthodontic records were provided by two private practices. 

Inclusion criteria encompassed patients with a unilateral or bilateral Class III canine relationship in the permanent dentition, with an edge to edge or anterior crossbite relationship which were deemed orthodontically manageable. All cases had initial and final dental casts and lateral cephalograms. 

Records with incomplete information, patients with more than one missing tooth per quadrant, patients with agenesis and/or syndromic patients, were excluded from the study. Models with chipped or broken teeth, or questionable articulation of upper and lower casts were eliminated. Patients with more than ten missed appointments or more than ten broken bracket/band during treatment, surgical cases, mini-implants, aesthetic orthodontic appliances and/or self-ligating appliances were also excluded. 

A sample size of 28 patients per group was deemed adequate based on a power of 80% and a bilateral alpha of 5%. A 6-month difference (20%) in treatment time between both the groups, assuming a standard deviation of 8 months was considered clinically relevant.^6^

After analysis of clinical records, 56 cases were included (Table 1). The sample consisted of 28 patients (13 women, 15 men) treated with Roth brackets, compared with 28 patients (14 women, 14 men) treated with Capelozza brackets. Both prescriptions had 0.022 x 0.028-in slots. The initial mean ages were 23.7 ± 10.81 years and 25.2 ± 10.72 years, respectively. 

**Table 1:** Sample description before treatment (T0).

	Roth			Capelozza III		
Orthodontists	Orthodontist A	Orthodontist B	Total (%)	Orthodontist A	Orthodontist B	Total (%)
Type of bracket	Mini-Ovation brackets (GAC International, Bohemia, NY, USA)	Synthesis brackets (“A” Company, San Diego, CA, USA)		Capelozza’s prescription III brackets (Abzil, São José do Rio Preto, Brazil)		
n	17	11	28 (50)	20	8	28 (50)
Angle canine classification						
Class III unilateral	10	7	17 (30.4)	10	4	14 (25)
Class III bilateral	7	4	11 (19.6)	10	4	14 (25)
Treatment type						
No nextraction	9	6	15 (26.8)	13	2	15 (26.8)
LR4 and LL4 extractions	4	0	4 (7.1)	3	1	4 (7.1)
Four first premolar extraction	1	0	1 (1.8)	2	0	2 (3.6)
Strippings	3	5	8 (14.3)	2	5	7 (12.5)
Sex						
Male	10	6	16 (28.6)	12	2	14 (23.2)
Female	7	5	12 (27.4)	8	6	15 (26.8)

Both orthodontists followed the same treatment protocols and used both types of brackets in the study. The following sequence of wires was used: 0.014, 0.016, 0.018 (NiTi), 0.020 steel (often here beginning lightweight 5/16-in Class III elastic), finishing with 0.019 x 0.025 steel. 

Cases treated with Roth brackets were finished between 1998 and 2005, while cases with Capelozza brackets, between 2003 and 2013. The oldest case included was started when both orthodontists had experience with at least 500 orthodontic finished cases.

Treatment duration was recorded in months. Furthermore, initial age, gender, number of appointments, missed appointments, amount of time during which Class III elastics were properly worn, and number of bond failures were also examined. Intervals longer than 45 days between visits were considered missed appointments. Extractions or missing teeth were confirmed using panoramic radiographs obtained before and after treatment. 

The skeletal characteristics of each group were evaluated from pretreatment lateral cephalograms. The analysis was performed using 3 linear (Wits, CoA and CoGn) and 6 angular (SNA, SNB, ANB, SNGo-Gn, IMPA and 1-PP) measurements.

Quantification of malocclusion was performed by PAR index (Peer Assessment Rating) applied to the initial (PAR T_1_) and final (PAR T_2_) dental casts.^13^ Measurements were obtained from the models by a calibrated single examiner and were reassessed within a 30-day interval, for error analysis. A digital caliper (model 530-102, Mitutoyo, Suzano, SP, Brazil) was used. 

The extent of the malocclusions correction as a result of orthodontic treatment was measured by the difference in percentages between the initial and final values ​​of the PAR index, applying the following formula:^14^

$$\%PAR\;\;=\frac{PAR\;T_{1}-T_{2}}{PAR\;T_{1}}x100$$

The rate of treatment efficiency is the ratio between the percentage of improvement and the treatment duration in months.^15^

Random error was found by Dahlberg’s formula^16^ and systematic error by the intra-class correlation test. D’Agostino-Pearson test was employed to examine normal data distribution. Comparison between groups was performed using MANOVA test. SPSS software for Windows, version 22 (IBM Corporation, SPSS Inc., Chicago, USA), was used, with a significance level of 5%.

## RESULTS

Random error was analyzed in order to measure PAR index and was found to be 1.2 (*p*> 0.05), with ICC equal to 0.99 (*p*< 0.0001), showing excellent repeatability. 

None of the cephalometric measurements as well as PAR index (*p*= 0.24) showed statistically significant difference between groups at T_1_ (Table 2).

**Table 2:** Mean and standard deviation (SD) for cephalometric measurements (SNA, SNB, ANB, SNGoGn, CoA, CoGn, IMPA, 1.PP) in Groups 1 (Roth) and 2 (Capelozza).

Cephalometric measurements at T_1_	Roth (n=28)		Capelozza (n=28)		p-value
	Mean	SD	Mean	SD	MANOVA
ANB	-0.4°	2.6	-0.8°	2.3	0.62
SNA	81.4°	4.7	82.1°	4.2	0.52
SNB	81.7°	3.9	83.0°	4.2	0.26
Wits	-4.4mm	3.0	-4.6mm	2.6	0.86
SNGo-Gn	33.5°	4.2	30.8°	5.7	0.06
CoA	92.9mm	7.2	92.0mm	7.6	0.65
CoGn	127.2mm	10.3	127.4mm	10.4	0.94
IMPA	85.3°	6.5	83.8°	6.6	0.41
1.PP	120.2°	6.7	120.3°	7.3	0.99

Both groups showed significant improvement in occlusion correction during treatment (95.79% for the group of patients treated with Roth brackets, and 92.15% for the group treated with compensation brackets, *p*< 0.001) with no difference regarding occlusal finishing obtained (PAR T_2_, *p*= 0.61, Table 3).

**Table 3:** Mean and standard deviation (SD) for initial age, treatment duration, number of appointments, PAR Index at T_1_ and T_2_, PAR Index improvement, treatment efficiency, missed appointments, bond or band failures, and Class III intermaxillary elastics time, in Groups 1 (Roth) and 2 (Capelozza).

Variable	Roth (n=28)		Capelozza (n=28)		p-value
	Mean	SD	Mean	SD	MANOVA
Initial age	23.75	10.81	25.28	10.72	0.59
Treatment duration (months)	33.15	11.19	26.19	9.10	0.01*
Number of appointments	29.5	8.0	25.6	8.2	0.07
PAR T_1_	28.85	13.55	24.92	11.17	0.24
PAR T_2_	1.92	2.01	2.21	2.23	0.61
% PAR improvement	95.79	10.66	92.15	10.16	0.22
Treatment efficiency	3.11	1.02	4.01	1.94	0.03*
Number of missed appointments	2.9	3.1	2.0	2.2	0.21
Number of bond failures	2.3	2.2	2.4	3.2	0.77
Class III elastic (months)	7.8	7.4	5.9	3.9	0.23

*p <0.05.

The mean treatment duration of Class III patients treated with the compensated Class III malocclusion brackets (Group 2) was 26.19 months, while in patients who used non-compensated brackets (Group 1) was 33.15 months. The difference was of nearly seven months (*p*= 0.01). 

By combining a faster orthodontic treatment and a similar occlusal finishing, present results showed that orthodontic treatment of Class III malocclusion using compensated brackets (median = 4.01) was more efficient (*p*= 0.03) than with non-compensated brackets (median = 3.11).

Missed appointments (*p*= 0.21), bond or band failures (*p*= 0.77), and number of months Class III intermaxillary elastics were worn (*p*= 0.23) were not significantly different between groups (Table 3). 

## DISCUSSION

For the orthodontist, the ability to more accurately predict treatment duration can be a valuable tool for practice management.^17^ It can help them to earn the patient’s trust.^18^ Furthermore, one should bear in mind that orthodontic treatment involves biological costs and longer treatments have been associated with root resorption.^19^

Our findings supports the claim^8^ that preadjusted orthodontic brackets designed for compensatory treatment of mild Class III malocclusion provide greater treatment efficiency compared to Roth preadjusted brackets - treatment efficiency being the ratio between the percentage of improvement and the treatment duration in months^15^. Despite Roth group being slightly better at “improvement in occlusion correction”, no differences regarding occlusal finishing were obtained between techniques. Significant clinical difference was found in treatment duration, that was about 7 months shorter in Capelozza III prescription.

The Peer Assessment Rating (PAR) was used to quantify the severity of the malocclusion given that it is a reliable^13^ and valid^20^ ^,^ ^21^ ^,^ ^22^ method that allows comparisons to be made between groups.^13^ The degree of improvement is organized into categories: “Worse - no different,” “Improved” and “Greatly improved.” It takes a reduction of 30% in PAR index in the outcome score for the treatment to be considered “Improved”, and above 30% to be considered “Greatly improved”.^13^ ^,^ ^20^ Both groups showed an improvement greater than 92.15%, indicating an excellent finishing. Because this was a sample of patients with mild Class III malocclusion - which as a rule involve a discrepant maxillomandibular relationship -, it is understandable that there should be high initial PAR index values ​​as well as a high percentage of improvement. 

Despite the fact that PAR index has been applied in several studies, it has some limitations. It fails to measure several important outcomes of orthodontic treatment, including: degree of orthodontically induced external root resorption,^19^ dental and facial aesthetic improvement,^17^ and, especially, patient satisfaction after orthodontic treatment.^18^ A prospective randomized study is suggested to cover these gaps and provide a deeper look into these variables.

Regarding the retrospective nature of this study, only consecutively treated cases were included. If the sample consisted of selected rather than consecutive subjects there would be a risk of unduly optimistic success rates.^23^ However, this is not a randomized clinical trial and as a retrospective study, it has some limitations. The difference in the time periods at which treatments were performed is one of them. The impact of time on the treatment efficiency has been previously published.^24^ A sample of 70 patients with Class I and Class II malocclusion treated by the same orthodontist during the same time period as the cases included in this study was considered. The aim was to determine whether or not there was a correlation between the year when treatment was started and the duration of orthodontic treatment. Thus, it would be possible to determine if a seasoned clinician could further hone their skills over the years and significantly decrease the time needed to treat their more recent cases. No statistically significant correlation was found (rs = -0.17, *p*= 0.32). 

An important issue is the age at the start of treatment, which was similar between groups. This standardization is important since as the patient grows older, a smaller improvement in the PAR index should be expected given that in adults the treatment goals may be limited by prosthetic rehabilitation.^22^ Furthermore, there is a clinical consensus that the dentoalveolar compensatory treatment of mild Class III malocclusions should be performed in adults or patients with no remaining significant mandibular growth changes.

Cephalometric data were not used as inclusion criteria. The main reason not to include them was to show that both groups had a similar baseline and that the skeletal discrepancies were not severe. Nevertheless, all patients included presented Wits value under -4.4 mm, confirming the sample as skeletal Class III patients, and the equivalency between groups. A Wits value of -5.0 mm had previously been quoted in the literature as the borderline for non-surgery Class III orthodontic treatment.^6^

Previous reports show that the number of missed appointments is a major factor that significantly affects treatment duration.^25^ It has also been reported that each bracket or band failure entails an additional 20 days of treatment time, and that patient cooperation appears to have a greater effect on duration of orthodontic treatment in mild Class III malocclusion patients. Having homogeneous samples (Table 2) was essential to ensure that the influence of the variable “bracket system” would be isolated compared to other factors that directly affect the duration of orthodontic treatment, such as: missed appointments, breakage and the use of intermaxillary elastics.^24^ ^-^ ^27^

It was previously claimed that the efficiency of compensatory orthodontic treatment depended substantially on biomechanical procedures capable of preserving or even strengthening bracket individualization^28^, i.e. the use of intermaxillary elastics. The results of this study do not support the assumption that a compensated bracket system implies in less intermaxillary elastic time wear since the two groups, despite the difference in treatment duration, used Class III intermaxillary elastics for the same amount of time. However, the Roth group presented a greater variability in the intermaxillary elastic wear time in treated cases, with a higher standard deviation. On the other hand, Class III elastic was introduced closest to the beginning of treatment in Capelozza group (median = 9 months) and later in patients from Roth group (median = 13 months). However, it is reasonable to believe that it can be due to the flaring of the anteroinferior teeth that are avoided when using compensated prescriptions, allowing for faster alignment and the earlier use of elastic.

Regardless of the size or form of the archwires to be used, angulation (tip) is expressed from the beginning of mechanics. This is not true for inclination, which can only be deployed with rectangular wires, by eliminating the play between the archwire and the bracket slot.^28^ Kattner and Schneider^29^ compared patients treated with Edgewise and Roth by two orthodontists. No significant differences were found. However, there were differences between the two clinicians: The clinician who had better occlusal results took longer to finalize and more often used full-sized arches (0.019 x 0.025-in) than the other practitioner. In our study, both orthodontists used steel 0.019 x 0.025-in archwires in their finishing stages, and no differences in occlusion were found after treatment. This outcome is similar to the findings of Moesi et al^30^ comparing treatments using Roth and MBT bracket prescriptions. It seems reasonable to believe that tooth movement promoted by the angulations built into the compensated brackets reduce roundtrip movements in the anterior region. As a result, optimal treatment efficiency is expected.

## CONCLUSIONS

Orthodontic brackets designed for dentoalveolar compensation of Class III malocclusions appear to be more efficient than non-compensated straight-wire prescription brackets in this sample. A prospective randomized study is suggested to cover the gaps of a retrospective study and provide a deeper look into the variables presented.

No difference in the quality of the final occlusal outcomes was observed, but treatment duration was shorter by seven months for patients treated with brackets designed for Class III malocclusion compensation. 
